# *Sensus Communis*: Some Perspectives on the Origins of Non-synchronous Cross-Sensory Associations

**DOI:** 10.3389/fpsyg.2019.00523

**Published:** 2019-03-07

**Authors:** Bahia Guellaï, Annabel Callin, Frédéric Bevilacqua, Diemo Schwarz, Alexandre Pitti, Sofiane Boucenna, Maya Gratier

**Affiliations:** ^1^Laboratoire Ethologie, Cognition, Développement, Université Paris Nanterre, Nanterre, France; ^2^STMS Ircam-CNRS-Sorbonne Université, Paris, France; ^3^Laboratoire ETIS, Université Cergy-Pontoise, Cergy-Pontoise, France

**Keywords:** cross-sensory, correspondences, infancy, development, perception

## Abstract

Adults readily make associations between stimuli perceived consecutively through different sense modalities, such as shapes and sounds. Researchers have only recently begun to investigate such correspondences in infants but only a handful of studies have focused on infants less than a year old. Are infants able to make cross-sensory correspondences from birth? Do certain correspondences require extensive real-world experience? Some studies have shown that newborns are able to match stimuli perceived in different sense modalities. Yet, the origins and mechanisms underlying these abilities are unclear. The present paper explores these questions and reviews some hypotheses on the emergence and early development of cross-sensory associations and their possible links with language development. Indeed, if infants can perceive cross-sensory correspondences between events that share certain features but are not strictly contingent or co-located, one may posit that they are using a “sixth sense” in Aristotle’s sense of the term. And a likely candidate for explaining this mechanism, as Aristotle suggested, is movement.

## Introduction

Everyday experience is multisensory. Even the simplest of activities entails the interaction of multiple sense modalities, and the sense modalities must operate together for perception of events to occur. How is it that our perception of objects, people and events is unitary and coherent? This question dates back to Aristotle (Suzuki, 1952) who proposed a common sense or “*sensus communis*” responsible for monitoring and coordinating the five senses out of which unified conscious experience arises. Indeed, Aristotle posited the *sensus communis* as a sixth sense; one that does not depend on specific sensory apparatus, but that is rooted in the possibility or potentiality of movement. Although great advances have been made in the fields of neuroscience, psychology and robotics in answer to Aristotle’s conundrum, the question of the origins and development of cross-sensory perception in infancy is still only partially answered. Specifically, a very limited number of studies have focused on the relations between sensory systems that convey similar or analogous information non-synchronously. A cross-sensory correspondence is the association between an attribute or dimension in one sense modality and an analogous attribute or dimension in another sense modality (Walker-Andrews et al., 1994). The way our brain integrates multimodal information and how this develops is still in debate. Nonetheless, many experiments and studies in the neurobiology of development show the importance of two major cerebral areas involved in cross-sensory integration, namely the superior colliculus (SC) and the parietal cortex (PC) (Stein et al., 2009). These two areas mature at different intervals and speeds before birth till the second-year and also represent multisensory information differently (e.g., egocentric vs. allocentric and reflexive vs. learned). Furthermore, they are not isolated circuits and work in combination with other regions in different sensorimotor loops depending on the types of behavior (automatic or rule-based), the timescale of learning and execution (short or long).

Considering the development of MI (multimodal integration) in the cortex, Keysers and Gazzola (2006) suggested that mirror neurons may be present at birth in the whole cortex. Heyes (2010) proposed that this mirroring mechanism could be based on the reinforcement learning of sensorimotor contingencies which assumes that visual (sensory) representations of action simultaneously seen and executed become linked to motor representations through Hebbian learning.

Many events involve perceiving the same property in different sense modalities. The size and shape of an object can, for example, be processed both visually and haptically, which leads to a unitary percept. Intensity of either light or sound can change over time and thus be perceived as a similar phenomenon. In fact, in everyday situations, contingent associations of different attributes simultaneously changing over time are experienced on a regular basis. A bouncing ball, for example, is perceived as regular up and down motion and as regular sound-silence alternation. Its movement generates a multimodal representation based on synchronous and analogous dynamic time variations in visual and auditory experience. Both the object’s attributes and its particular qualities of motion are thus represented.

One of the first experiences of a newborn infant is of a person talking to him/her, involving both synchronous and non-concurrent analogous changes and shifts in the contours of facial, vocal, postural, and tactile stimulations. Yet, very little is known today about the underlying processes by which very young infants associate analogous expressions occurring non-synchronously across modalities. For example, when the mother raises her eyebrows and then, less than a second later, produces a higher pitched rising-falling utterance, does the infant associate the expressive contours of her behavior across modalities? Infant-addressed behavior is usually exaggerated compared to adult-addressed expression. Furthermore, in social infant-directed expression, synchronous behavior in various modalities fosters high degrees of intersensory redundancy, which is thought to facilitate perception for young infants (Bahrick and Lickliter, 2002). An important body of knowledge has been gathered on the perception of synchronous changes in different modalities by young infants (Bremner et al., 2012). Much less research has been concerned with non-synchronous but analogous changes over time and across different sense modalities.

Some studies in adults have shown that stimuli presented non-synchronously to different sense modalities are perceived as having correspondences when they are matched on certain features such as intensity or duration. More surprisingly, adults also perceive stable correspondences between certain object attributes that are not straightforwardly connected. For example, adults readily associate higher pitch in sound with angularity of shape or acidity in taste and smell (Crisinel and Spence, 2009; Deroy et al., 2013). The existence of these types of correspondences raises important questions. Can these correspondences be considered as analogies? Are they learned through experience? Are they automatic? Are there different kinds of cross-sensory correspondences? When do infants make correspondences between static cross-sensory attributes, between cross-sensory events that change together over time and between cross-sensory events that share certain features but do not co-occur? Do some types of cross-sensory correspondences develop before others? Answering these questions could provide insight into the nature and development of abstraction abilities. The present review will focus on: (1) the development of cross-sensory associations in the first year and (2) its possible role in language development.

## Cross-Sensory Associations in the First Year of Life

In the past 20 years or so, the study of cross-sensory perception has largely been based on a developmental model of progressive integration of the senses. This enquiry has intensified in recent years in the broad field of cognitive science. Much research has focused on how adults integrate information perceived in different sense modalities. Although everyday events are perceived as unitary, they are usually thought to involve the integration of information processed independently by each sensory system. Multimodal knowledge would be acquired through repeated experiences of such intersensory integration, which is extended to new situations. For example, large heavy objects usually make deep and loud sounds when they fall. This repeated association may orient representations based on a unimodal experience, so that a loud sound can conjure up a representation of a large object in the absence of visual input. Implicit multimodal knowledge may also be obtained from perceived correspondences between non-concurrent events. This applies particularly for perception of animate beings where multimodal knowledge could be driven by basic principles of sociality. Thus, the fact that young infants are highly motived toward social stimuli, and spend much more time in contact with persons than with objects, might explain why they rapidly learn to associate sensory experience in a given modality with sensory experience in other modalities. It is also possible that infant’s readiness for social interaction is linked to a precocious ability to perceive correspondences between modalities.

The use of multimodal knowledge is therefore a fundamental ability, enabling the identification of events and adaptive responses to these events (Simon, 2008). Many studies investigating the behavioral effects of multisensory experience have shown the benefits it affords (Press et al., 2004; Lippert et al., 2007) and it appears quite clearly to facilitate and optimize learning (Seitz et al., 2006). Yet, the development of such capacities is still unclear. One hypothesis formulated by researchers, the early integration hypothesis, proposes that cross-sensory integration is already present from birth while another hypothesis, the late integration hypothesis, emphasizes the role of experience in the development of cross-sensory associations (Dionne-Dostie et al., 2015). Here, we will focus on how infants sense their world and how they make sense of it, rather than on how the senses operate alone and together in infants. In particular, we will distinguish studies on cross-modal integration from studies on cross-modal transfer.

### Studies on the Precocity of Cross-Sensory Integration

Although the literature on cross-sensory integration in infants is fairly recent, the idea that this capacity emerges very early during development is not new. According to Gibson’s (2014) ecological theory of perception, amodal information, that is information not specific to any one modality and that can be conveyed redundantly across many senses, is directly obtained from adaptive interaction between organisms and their environments. Duration, spatial extent, temporal synchrony, shape, and intensity are considered to be important amodal characteristics of objects and events. Gibson (1969) proposed that amodal spatial and temporal dimensions are available to all sensory modalities already from birth.

Based on a large body of experimental research on the role of these amodal characteristics in early perceptual abilities, Bahrick and Lickliter (2000) put forward the “intersensory redundancy hypothesis” to explain how infants perceive coherent, unified multimodal objects and events through different sense modalities. This theory proposes that, in order to be perceptually integrated, the same information must be spatially coordinated and temporally synchronous across two or more sensory modalities and that cross-sensory integration is thus only possible for amodal properties that are not specific to a single sense modality (e.g., shape, rhythm, duration, and intensity). In other words, regardless of which sensory modality is solicited, similar qualities are perceived through the integration of information from diverse sources. For instance, the sound and sight of a bouncing ball are integrated because auditory and visual information is synchronous (shares a common tempo and rhythm) and originates from the same location. Therefore, sensitivity to amodal properties allows young infants to selectively direct their attention to unitary and meaningful events in their environment (Bahrick, 1992).

Prefiguring Bahrick and Lickliter’s hypothesis, Lewkowicz and Turkewitz (1980) were the first to demonstrate that 3-week-old infants can match sound and light intensities. In their seminal study, infants exposed to light spots of different intensities looked preferentially toward a light of intermediate intensity. However, infants first exposed to sounds at various intensity levels and then to light spots of similar intensities preferred the light spot that matched the sound stimulus. Lewkowicz and Turkewitz (1980) concluded from these results that young infants attend to quantitative variations in stimuli. It has also been shown that newborns are able to learn arbitrary auditory-visual associations (e.g., between an oriented colored line and a syllable), but only when the visual and auditory information are presented synchronously (Slater and Kirby, 1998). Furthermore, newborn infants are able to associate objects and sounds on the basis of temporal synchrony (Slater and Kirby, 1998). They can also associate a vocal sound from a non-human primate with the corresponding lip shape of the primate’s face (Lewkowicz et al., 2010) based on temporal synchrony.

Some authors have proposed that the results of these studies can be explained by the importance of synchrony for perception in the first months of life (Bahrick, 1987; Lewkowicz, 1996; Bahrick and Lickliter, 2012). According to this view, early cross-sensory integration would mainly be based on temporal synchrony (e.g., a sound and an image occurring together), and spatial colocation (e.g., coincidence of the location of a sound and an image). Quite paradoxically however, according to this view, an infant would not be surprised to perceive his/her mother’s face with a male voice as long as the lips’ movements are temporally synchronous with the mouth movements.

While temporal synchrony has been recognized as a fundamental dimension for establishing the link between visual and auditory information about an event or an object, it does not appear to be necessary for infants to integrate and make sense of multimodal events. For example, Izard et al. (2009) showed that newborn infants spontaneously associate visuo-spatial arrays of objects with auditory sequences of events based on similarities in numerosity. Despite the absence of synchrony between the objects and the sounds, newborns were able to respond to abstract numerical quantities presented across these two modalities (i.e., auditory and visual). Guellaï et al. (2016) have also shown that newborns make accurate audio-visual associations based on the non-synchronous presentation of stimuli. Infants were presented with two dynamic facial displays uttering two different sentences but where only one of them corresponded to one of the two displays. Both facial displays started and stopped at the same time. Thus infants used cues other than temporal synchrony to match utterances to the corresponding facial movements.

As already stated, the main studies in the literature have shown that infants are able to integrate information from different sense modalities very early on, and primarily based on temporal synchrony. Nonetheless, in everyday social situations, many multimodal events can be considered as sharing common features that occur non-synchronously. For example, the infant can be tickled by his/her mother, and a few seconds later she can say something that prosodically matches her gesture. In order to perceive such an event as unified and to make sense of it, infants must transfer information from one modality (proprioception) to other modalities (audio-visual). Therefore, cross-modal transfer must be an important ability in early infancy.

### Studies on the Precocity of Cross-Modal Transfer

Studies on the cross-modal transfer of information from touch to vision have revealed that neonates are able to process and encode shape information about haptically explored objects and to discriminate between subsequently presented visual objects (Streri and Gentaz, 2004). Newborns are also able to visually recognize a texture that they previously touched and to tactually recognize the texture that they previously saw even though they are presented sequentially (Sann and Streri, 2008). Meltzoff and Borton (1979) pioneering study reported that 1-month-old infants show a clear visual preference for objects with which they had been familiarized through oral presentation. Thus, a handful of studies have shown that transfer of information is possible from haptic experience to vision already at birth. In everyday life, infants explore the world haptically and also experience being touched by other people during social interactions and care practices. However, very little is known to date about infants’ perception of *being* touched and how that particular kind of information could be transferred to other sensory modalities such as vision and audition. One recent study suggest that newborns are capable of associating tactile stimulation on their own body with a congruent visual image of the same tactile stimulus (Filippetti et al., 2015).

In early social engagement with an adult, infants probably experience non-synchronous yet contingent expressions in different modalities. Therefore it is likely that infants are capable of integrating matching non-synchronous events as well as synchronous ones. Nevertheless, it remains unclear why cross-sensory matching abilities are present so early in life. One possibility is that such a process allows infants to perceive invariant aspects of the environments they must rapidly adapt to. Moreover, it has important implications for the development of face and emotion recognition. Studies using a visual preference paradigm in a multimodal context for human faces have reported that as early as 2 months of age, infants can associate phonetic information from voices with lip movements (Kuhl and Meltzoff, 1982; Patterson and Werker, 1999, 2003). More recently, it has been shown that 8-month-olds can associate a speech stream with the corresponding facial movements even when the speech stream is low-pass filtered (Kitamura et al., 2014), suggesting that prosody can be perceived both in the auditory and visual modalities (Esteve-Gibert and Guellaï, 2018). In addition, 4-month-old infants can perceive affect (joy, sadness, or anger) in speech sequences that are supported by audio-visual presentations of faces (Walker-Andrews and Lennon, 1991; Flom and Bahrick, 2007).

Overall, these studies support the idea of a precocious cross-sensory perception capacity, based on both cross-modal transfer and cross-sensory integration. The underlying mechanisms and the development of this capacity is still puzzling and remain largely unknown. Are general associative learning mechanisms sufficient to explain how infants come to pair sensory cues across modalities, or do specific learning processes or constraints guide the acquisition of some (or all) cross-sensory correspondences? Studies using neuroimaging techniques could help identify the underlying mechanisms of cross-sensory perception. Furthermore, research on prenatal perception suggests that at birth infants have some experience of multisensory events. Studies on premature babies could provide compelling data to help understand the foundations and mechanisms of cross-sensory associations. Experimental studies of cross-sensory perception suggest that sense modalities operate from the earliest days of life.

Evidence for the existence of not straightforwardly redundant cross-sensory integration in infants (0 to 12 months old) and toddlers (12 to 24 months old) has begun to be gathered. Cross-sensory correspondences between features of speech sounds and visual shapes have been demonstrated as early as 4-months of age (Peña et al., 2011; Ozturk et al., 2013). Similarly to adults, infants associate specific shapes to particular linguistic sounds. Why do such associations exist? In the next section, we will focus on sound symbolism as a specific form of cross-sensory association that could be an important starting point for the development of language.

## The Particular Case of Sound Symbolism

One of the most amazing capacities of our species is its ability to combine symbols in order to communicate specific meanings (Deacon, 1997). This capacity underlies the emergence of all natural languages. While the phylogeny and ontogeny of this skill have been the center of interest of many researchers in the last decades, they have raised vast and as yet unresolved debates across disciplines. For example, there is no clear account of how the human mind came to support a symbolic system that is largely disconnected from direct perception in the first place. In the “Cratylus,” Plato describes Hermogenes as stating “if one substituted one name for another, the latter would be as correct as the former” to which Cratylus answers that there is “for each object a name that is its own and that belongs to it intrinsically, or by its nature.” Socrates then concludes that even if in general the link between a thing and its name is arbitrary, there are, nevertheless, some noble words whose sound reflects their meaning. Yet one of the founders of modern linguistics, De Saussure (1989), proposed that all linguistic symbols are arbitrary, in other words, that there is no natural connection between linguistic form and linguistic meaning.

Even if the later proposition is the dominant view in linguistics today, a number of scholars have noted and studied the occurrence of analogical or iconic relations between words and their references. Some linguists and psychologists (Köhler, 1929; Sapir, 1929) have provided empirical evidence of non-arbitrary links between the signifier and the signified. The idea is that certain sounds are meaningful in themselves. This idea was first proposed by Gestalt psychologists, and is known as “sound symbolism.” One of the most famous experiments on this question was proposed by Köhler (1929). Using a simple forced choice task, adult participants were given two nonsense words, *maluma* and *takete*, and two abstract shapes, a rounded shape and an angular one. They were asked to match the words to the shapes. Kohler’s striking result was that almost all subjects identified the rounded shape as the *maluma* and the angular one as the *takete*. These findings were the first to show that linguistic sounds can bear an indirect yet natural connection to their referents. Furthermore, this connection is distinct from linguistic sound imitation, or sound analogy which involves association within the same modality. The Köhler task demonstrates a cross-sensory association between two sensory domains, vision (i.e., shape) and sound (i.e., spoken word).

In the past decades Kohler’s naming bias has been replicated cross-culturally (Bremner et al., 2013) and with various stimuli (Knöferle and Spence, 2012; Hanson-Vaux et al., 2013). Ramachandran and Hubbard (2001) used different non-word and visual stimuli in their version of Köhler’s experiment. Their words, *bouba* and *kiki* and the term ‘bouba kiki effect’ are now well-known in the literature. In their experiment, the rounded shape is consistently named as *bouba* and the angular shape as *kiki*.

Most studies to date on sound symbolism have explored the phenomenon in adult populations. Though the results are quite striking, it is not clear to what extent sound symbolism is dependent on experience. What about infants? One way to resolve this issue is to explore it developmentally, through infancy.

It is known that already from birth infants are sensitive to congruency between audiovisual speech inputs (Guellaï et al., 2016). Nevertheless, only a handful of studies have investigated the emergence of sound symbolism. So far, studies on infants have explored only associations between vision and audition (Peña et al., 2011; Maurer et al., 2013; Ozturk et al., 2013; Imai and Kita, 2014), and, moreover, they present divergent results. Notably, some studies have shown the bouba-kiki effect as early as 4 months (Peña et al., 2011; Ozturk et al., 2013) but these findings have been undermined by other studies that did not find this effect in infants (Fort et al., 2013).

Interestingly, recent studies have shown that this bias is not limited to the auditory-visual domains in adults. It has been found across different sensory modalities such as taste and sound (Knöferle and Spence, 2012) or odor and vision (e.g., a “sweet” odor is associated with a rounded shape and an “acid” one with a spiky shape) (Hanson-Vaux et al., 2013). The reason for such biased associations remains largely unknown but some possible explanations can be proposed. One possibility is that these biases, which are grounded in perception, played an important role in the evolution of the human language capacity. This possibility has received only little interest so far. Some authors proposed that these associations supported the emergence of a small-scale communication system, or protolanguage, mainly based on non-arbitrary associations, from which fully symbolic languages emerged (Cuskley, 2013). How might a non-arbitrary protolanguage have become arbitrary? One way to answer this question is to adopt an ontogenetic perspective and to investigate sound symbolism at different stages of early language development, exploring various types of cross-sensory correspondences across all sense modalities (Walker, 2016).

## Conclusion and Perspectives

The present review aimed at presenting a state of the art of studies on cross-sensory perception. It bridges a gap between studies focused on cross-sensory integration which show that spatio-temporal co-occurrence is a crucial context for infants to associate events in different sense modalities and other studies showing that even without strict co-occurrence very young infants can associate events in different sense modalities. These two areas of research together thus suggest that infants are equipped from birth to make sense of their environments through integration and transfer of experience between sense modalities. Yet these lines of research do not satisfactorily inform us on Aristotle’s suggestion of a “*sensus communis*” and further studies are needed to understand if and how young infants are able to associate non-synchronous events in different sense modalities. Indeed, if infants can perceive cross-sensory correspondences between events that share certain features but are not strictly synchronous or co-located, one may posit that they are using a “sixth sense” in Aristotle’s sense of the term. And a likely candidate for explaining this mechanism, as Aristotle suggested, is movement. Indeed, some researchers have begun to highlight the existence of strong overlap in brain structures involved in the perception of vision, sound and touch together and in motor control and planning (Gallese and Lakoff, 2005). Starting *in utero*, body movement may thus be a crucial process for developing a multi-modally integrated brain. Furthermore, if infants are able to perceive seemingly complex correspondences, between analogous but non-identical phenomena, one may assume that they are well equipped to make sense of the complex expressions of their social partners and to respond with an equally sophisticated preverbal sense-making ability.

Few studies to date have explored the development of these different types of cross-sensory correspondence longitudinally. An interesting approach would be to use developmental robotics to build and test models of cross-sensory perception pathways. The field of developmental robotics looks toward infant development for inspiration, data, and guidance, in order to build models of learning that may be useful for a better understanding of typical and atypical human development of cross-sensory experience. Recently, Brunetti et al. (2018) evidenced that pitch/size correspondence in adults was relative in nature, that is, adults match sound pitch to the corresponding image size depending on number of trials. Therefore, the role of experience in the development of cross-sensory integration is still unclear. The field of developmental robotics is motivated by the construction of autonomous robots and also by the idea of using the robot as a tool to investigate cognitive models. For example, Thomaz et al. (2005) used a robot head named “Kismet” which was able to: recognize gestures (pointing gestures) and facial expressions; with a head-mounted tracker to evaluate a human’s object of attention; and to respond to vocal stimuli. These types of applications in the field of developmental robotics could help understanding the role of experience in cross-sensory associations.

## Author Contributions

All authors listed have made a substantial, direct and intellectual contribution to the work, and approved it for publication.

## Conflict of Interest Statement

The authors declare that the research was conducted in the absence of any commercial or financial relationships that could be construed as a potential conflict of interest.
